# The G1 phase optical reporter serves as a sensor of CDK4/6 inhibition *in vivo*

**DOI:** 10.7150/ijbs.52101

**Published:** 2021-01-31

**Authors:** Cuiping Guo, Yuxian Guo, Jingjing Liu, Yiyang Gao, Min Wei, Ruijun Zhao, Min Chen, Guojun Zhang

**Affiliations:** 1 Cancer Center & Department of Breast and Thyroid Surgery, Xiang'an Hospital of Xiamen University, School of Medicine, Xiamen University, Xiamen, Fujian, China.; 2 Cancer Research Center, School of Medicine, Xiamen University, Xiamen, Fujian, China.; 3 Clinical Central Research Core and Key Laboratory for Endocrine-Related Cancer Precision Medicine of Xiamen, Xiang'an Hospital of Xiamen University, Xiamen, Fujian, China.; 4 The Breast Center, Cancer Hospital of Shantou University Medical College, Shantou, Guangdong, China.; 5 Guangdong Provincial Key Laboratory for Breast Cancer Diagnosis and Treatment, Cancer Hospital of Shantou University Medical College, Shantou 515041, China.; 6 Present address: Department of Thyroid and Breast Surgery, The Eighth Affiliated Hospital, Sun Yat-sen University, Shenzhen, Guangdong, China.

**Keywords:** CDK4/6, bioluminescence, cyclin E, the G1 phase of the cell cycle, non-invasive molecular imaging

## Abstract

Visualization of cell-cycle G1 phase for monitoring the early response of cell cycle specific drug remains challenging. In this study, we developed genetically engineered bioluminescent reporters by fusing full-length cyclin E to the C-terminal luciferase (named as CycE-Luc and CycE-Luc2). Next, HeLa cell line or an ER-positive breast cancer cell line MCF-7 was transfected with these reporters. In cellular assays, the bioluminescent signal of CycE-Luc and CycE-Luc2 was accumulated in the G1 phase and decreased after exiting from the G1 phase. The expression of CycE-Luc and CycE-Luc2 fusion protein was regulated in a cell cycle-dependent manner, which was mediated by proteasome ubiquitination and degradation. Next, our *in vitro* and *in vivo* experiment confirmed that the cell cycle arrested by anti-cancer agents (palbociclib or 5-FU) was monitored quantitatively and dynamically by bioluminescent imaging of these reporters in a real-time and non-invasive manner. Thus, these optical reporters could reflect the G1 phase alternation of cell cycle, and might become a future clinically translatable approach for predicting and monitoring response to palbociclib in patients with ER-positive breast cancer.

## Introduction

Extensive clinical evidence warrants targeting cell cycle as a therapeutic approach for cancer. Discovery of cell cycle-specific anti-cancer drugs involves several major processes including target identification and validation, high-throughput screening, lead optimization, and finally preclinical and clinical trials 1. One of the key objectives of drug discovery is to evaluate whether a compound can hit the target if it alters downstream molecular pathways in cultured cells and living animals. Traditionally, in animal studies, target validation is performed invasively by immunohistochemistry and/or molecular profiling after dissection of targeted organs/tissues 2. However, non-invasive reporter imaging approaches not only provide a longitudinal and temporal pharmacodynamic readout in the same group of animals but also measure real-time dynamic changes in drug targets. Among the MR imaging, nuclear imaging, and optical imaging technologies 3, optical imaging is based on quantitative or qualitative changes in light emission by fluorescent or bioluminescent proteins.

Many pharmaceutical companies are currently developing cell cycle-specific drugs as potential anti-cancer agents such as 5-FU and potent and selective CDK (2, 4/6, 7, and 9) inhibitors. Treatment of cells with 5-FU leads to the accumulation of cells in the S-phase, and treatment of cells with CDK2 or CDK4/6 inhibitors prevents the phosphorylation of tumor suppressor RB, thereby invoking cancer cell cycle arrest in the G1 phase. Recently, due to the striking clinical trial results of the CDK4/6 inhibitors demonstrating substantial improvements in progression-free survival 4-8, these inhibitors have transited rapidly from preclinical studies to the clinical arena, and three of them (palbociclib 6, abemaciclib 9, 10 and ribociclib 11) have already been approved for the treatment of advanced, estrogen receptor (ER)-positive breast cancer patients. In the clinic, the effect of cell cycle-specific anti-cancer drugs on decreasing tumor burden has been assessed by the response evaluation criteria in solid tumors (RECIST) 12. However, the reduction in tumor size can take several weeks to become manifest and, in some cases, may not occur at all. Thus, scientists have been focused on the development of molecular imaging methods to distinguish responders versus non-responders at early time points.

The cell cycle is regulated by both intracellular and extracellular signals. The transition from M to G1 phase during cell division could be determined by morphological changes. Mostly, G1/S transition is observed after nuclear bromodeoxyuridine (BrdU) staining. Additionally, fluorescent protein engineering and live-cell imaging techniques have fueled the concurrent development and application of genetically encoded fluorescent reporters for tracking the different phases of the cell cycle. For example, fluorescent indicators for S phase and the subsequent transition to G2 in live cells have been developed by fusing a fluorescent protein to proliferating cell nuclear antigen (PCNA) 13 or the C terminus of helicase B (GE healthcare) 14. Moreover, in the widely used Fucci reporter system, G1-phase cells are labeled with a fluorescent protein fused to Cdt1, while cells in S, G2, or M are labeled with another fluorescent protein fused to Geminin 15, 16. However, since the identification of cell-cycle transitions requires the detection of subtle and often minute changes in the distribution pattern and intensity of fluorescence signals, these markers cannot track phase transitions with high contrast. On the contrary, novel bioluminescent imaging systems based on luciferase could modify the substrate *in vivo* and in so doing produce light, which can be detected using sensitive cooled charge-coupled device cameras. The advantage of bioluminescence over fluorescence imaging is that the sensitivity for detecting signal is very low (10^-17^-10^-15^ M) with no imaging background. Furthermore, this technique could offer a non-invasive way for rapid, real-time monitoring of biological events in living cells 17 and animals 18, 19. Thus, as an alternative to fluorescence assay, bioluminescence imaging is capable of ironing out the flaw of tissue autofluorescence resulting in high signal to noise ratio and provides complimentary advantages for preclinical applications *in vivo.*

The firefly luciferase (Luc) protein is the most widely used bioluminescent imaging reporter for monitoring the status of proteins i.e., p53 protein 20 or NFκB activity 18, 21 or the proteasome inhibition 22-24. The key proteins that drive cell cycle progression have been selected and developed as optical reporters for different cell cycle phases based on their specificity, sensitivity, and versatility, in combination with the non-invasive and non-destructive nature of the bioluminescent imaging and the power of genetic encoding. These proteins are mainly being studied to determine whether they are uniquely suited for monitoring the therapeutic response. So far, our research group has developed a series of bioluminescence reporters to visualize cell cycle changes by detecting the accumulation of cell cycle proteins and used these tools to monitor the treatment response of cell-cycle specific drugs. For example, p27-Luc was downregulated when cell cycle was arrested in late G1 or S phase, and the non-invasive bioluminescent imaging of this reporter was observed as it accumulated after treatment of CDK2 inhibitors (flavopiridol and R-roscovitine) 25. Similarly, cyclin A2-luciferase was used to monitor S-phase arrest by the drug 10-hydroxycamptothecin (HCPT) 26. Thus, the bioluminescence imaging reporters are helping to bridge the gap between our understanding of critical biologic events and the clinical applications of specific cell cycle inhibitors.

Cyclin E is expressed from the mid G1 phase through late G1 phase and degraded in S phase by proteasomal ubiquitin-dependent proteolysis. Cyclin E promotes the cell cycle progression and drives cells into the S phase by activating CDK2. In the present study, we harnessed this feature of cyclin E to develop the cyclin E and Luc fusion proteins as genetically encoded indicators for the G1 phase. The application of the G1 phase reporter for monitoring or predicting the early response of cell-cycle-specific drugs might help to identify patients who are most likely to benefit from these drugs alone or in combinational-therapy. The preclinical study described herein was designed to test the feasibility of this approach.

## Materials and Methods

### Antibodies, chemicals, animals and siRNA

Mouse polyclonal antibody against cyclin E and mouse polyclonal antibody to Fbw7 and β-actin were purchased from Santa Cruz Biotechnology (Santa Cruz, USA). Several Chemicals were purchased from commercial companies: Nocodazole and MG132 from Sigma (St. Louis, USA), palbociclib from MedChemExpress, D-luciferin from Cold Spring Harbor, and 5-FU from Tianjin Jinyao Amino Acid co. LTD. Nu/Nu nude mice 6-8 week-old were purchased from Beijing Vital River Laboratory Animal Technology Co. Ltd. siRNAs were synthesized by GenePharma (Shanghai, CN). siRNA sequences were unique to their intended targets based on BLAST searches. The sequence of siRNA specific to Fbw7 were 5'-AACCUUCUCUGGAGAGAGAAAUGTT-3' (Sense), 5'-CAUUUCUCUCUCCAGAGAAGGUUTT-3' (Anti-Sense). The scramble siRNA sequences were 5'-UUCUCCGAACGµGUCACGUTT-3' (Sense), 5'-ACGµGACACGUUCGGAGAATT-3' (Anti-Sense).

### Construction of plasmids

**CycE-Luc plasmid:** Human full-length cyclin E was amplified by PCR using a primer pair (Forward: 5'-CAGGATCCCCAAGCTTCCATGAA GGAGGACGGCGGCGC-3'; Reverse: 5'-CCGGAATGCCAAGCTTG CGCCATTTCCGGCCCGCTGC-3'). The PCR fragment of cyclin E was digested with Hind III restriction enzyme and ligated into an expression plasmid 10-4 cyclin E promotor (Addgene, Sidney St., Cambridge, MA, #8458), in which firefly luciferase cDNA is under control of the cyclin E promoter. The cloned expression construct for cyclin E and Luciferase fusion protein was verified by automated DNA sequencing (Sangon Co. Ltd, Shanghai, China).

**CycE-Luc2 plasmid:** Similarly, human full-length cyclin E was amplified by PCR using a primer pair (same forward and reverse primers as CycE-Luc plasmid construction). The CycE cDNA fragment was cloned into the pcDNA3.1(+)/Luc2=tdT vector (Addgene, Sidney St., Cambridge, MA), in which firefly luciferase 2 cDNA is under control of the CMV promoter. The cloned expression construct for cyclin E and luciferase 2 fusion protein was verified by automated DNA sequencing (Sangon Co. Ltd, Shanghai, China).

### Transfection of cyclin E and Luc fusion plasmids in cell lines

HeLa cells and MCF-7 cells were maintained in Dulbecco's Modified Eagle Medium supplemented with 10% fetal bovine serum and 1% penicillin/streptomycin. Plasmid transfections were performed with Lipofectamine 2000 (Invitrogen, Camarillo, USA) according to the manufacturer's instructions. To establish stable cell lines expressing cyclin E and Luc fusion proteins, cells were transfected with expression vectors. Forty-eight hours later, cells were exposed to 1000 μg/mL G418 (Gibco, USA). After 14 days of selection, G418-resistant clones were randomly picked and cultured in the medium containing 500 μg/mL G418. Single clonal cell lines were established with single-cell deposition into each well of the 96-well plate.

### The transfection procedure of siRNA

Cells were seeded in 6-cm dishes and transfected with siRNA targeting Fbw7 using Lipofectamine 2000 transfection agent (Invitrogen, Camarillo, USA) according to the manufacturer's recommendations. Scramble siRNA was used as a negative control for comparison. All siRNAs were transfected at 100 nM final concentration. The transfection reagent was replaced by complete medium after incubation for 6 h, and cells were collected 48 h post transfection for western blot analysis.

### Cell cycle synchronization

**Nocodazole block**: Cells were cultured in regular media for 24 h to achieve cell adherence before fresh culture medium containing 0.4 μg/mL nocodazole (Sigma, St. Louis, MO, USA) was added, and then cells were further incubated for 18 h.

**Double thymidine block**: Following overnight adherence, the culture medium on cells was replaced with fresh medium supplemented with 2 mmol/L thymidine (Sigma, St. Louis, MO, USA) and incubation continued for 14 hours. The monolayers were then washed with PBS and incubated for 12 hours in fresh medium. At this point, the medium was again replaced with fresh medium containing 2 mmol/L thymidine and incubation continued for a further 14 hours.

### Cell cycle analysis

Cells were fixed with ice-cold 70% ethanol at 4 °C overnight in dark, then centrifuged and incubated in 0.5ml staining buffer containing 10 µl propidium iodide and 10 µl RNase A (YEASEN, Shanghai, China) for 30 min at 37 °C in dark. DNA contents were analyzed using a FACScan flow cytometer. DNA histograms were obtained using ModFit LT 3.1 (Verity Software House, Shanghai, China).

### Bioluminescent imaging *in vitro* and *in vivo*

For *in vitro* studies, D-luciferin was added to tissue culture medium, to a final concentration of 150 µg/ml. Five minutes later, photons were counted using the IVIS imaging system (Xenogen) according to the manufacturer's instructions. Data were analyzed using Living Image software (version 4.5.5, Xenogen).

### Western blot analysis

Proteins from cultured cells were extracted by lysis in Reporter Lysis Buffer (Promega) and quantitated by BCA protein assay (Pierce Biotechnology). Equal amounts of protein per lane (30 µg) were subjected to SDS-PAGE, transferred to nitrocellulose membranes, and immunoblotted with antibodies against cyclin E, β-Actin, GAPDH and Fbw7. Protein bands were developed using horseradish peroxidase-conjugated secondary antibodies (Santa Cruz Biotechnology) and visualized using ECL detection reagents (Pierce Biotechnology) according to the manufacturer's instructions. Cells were lysed with lysis buffer (Promega). The supernatants of cell lysates were separated by 10% SDS-PAGE and proteins were transferred to polyvinylidene difluoride membrane. CycE-Luc2 and CycE-Luc fusion protein expression were detected by using antibodies against Cyclin E (Santa Cruz) using β-actin as a control.

### Luciferase assay *in vitro*

Luciferase activity in cell extracts was assessed using the Luciferase assay system (Promega) according to the manufacturer's instructions. Briefly, cells were lysed by rocking in passive lysis buffer (Promega) for 15 min at room temperature. For each assay, 10 microliters of cell extract were used for measuring luminescence intensity after administration of D-luciferin using a Lumat LB9507 luminometer (Berthold Technologies).

### *In vivo* mouse imaging experiments

All experimental procedures with animals used in this study were approved by the Association for Assessment and Accreditation of Laboratory Animal Care International (AAALAC) and the Institutional Animal Care and Use Committee (IACUC) of Shantou university medical college, China (SUMC2018-306). Approximately 1×10^7^ monoclonal HeLa cells producing CycE-Luc in 0.2 mL PBS were injected subcutaneously into sites on the right flank of Nu/Nu nude mice under anesthesia (Sodium pentobarbital). For *in vivo* studies, at 0, 24, and 48 h after intraperitoneal injection of PBS, MG132 (2 mg/kg per mouse), and 5-FU (25 mg/kg per mouse), mice were administered D-Luciferin (150 mg/kg) and imaged using the Xenogen IVIS Lumina imaging system (Xenogen/Caliper) as described before.

Alternatively, Nu/Nu nude mice were randomized, implanted s.c. with CycE-Luc2 expressing monoclonal cells in the right axilla. Palbociclib treatment was initiated when tumors reached ~ 100 mm^3^ by oral gavage daily at 150 mg/kg for 8 days twice a week after implantation, and the mice were imaged using the Xenogen IVIS Lumina imaging system.

In the end, mice were necropsied with tumor flash-frozen for molecular and histological studies. Data were analyzed using Living Image software.

### Immunohistochemical analyses

Mice were euthanized and tumor tissues were collected 48 h after intraperitoneal administration of PBS, MG132 (2 mg/kg per mouse), and 5-FU (25 mg/kg per mouse). Samples of tissues processing and immunohistochemistry staining were performed as previously describe 27. The expression of cyclin E in tumors was detected using goat polyclonal antibody for cyclin E (R & D, 1:100 dilution).

### Statistical analysis

The data significance was evaluated by SPSS 11.5 software. All values were presented as mean ± SD. Statistical significance among various groups was calculated by one-way ANOVA using post hoc multiple comparisons, when p<0.05 was considered statistically significant.

## Results

### Construction of CycE-Luc reporter

**Figure 1:**
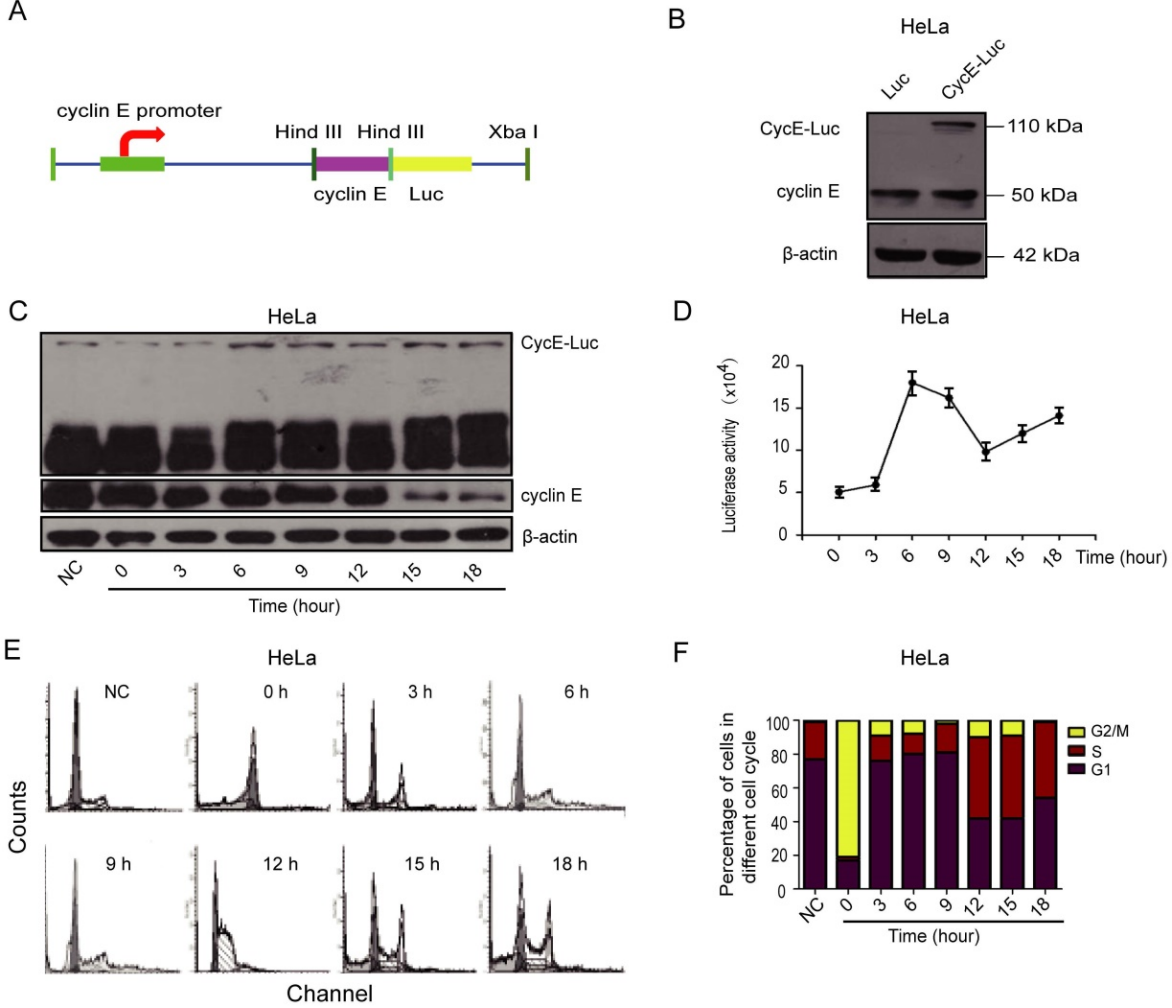
** Characterization of bioluminescent CycE-Luc reporter for the G1 phase.** (A) Schematic diagrams of CycE-Luc constructs. The recombinant plasmid CycE-Luc encoding cyclin E-Luc fusion protein contained the cyclin E gene fused in-frame at the N termini of luciferase gene under the control of the cyclin E promoter. (B) CycE-Luc fusion protein was expressed in transfected HeLa cells by immunoblotting. (C-F) HeLa-CycE-Luc cells were first blocked in M phase by adding 0.4 µg/mL Nocodazale overnight. After removing Nocodazale to release G2/M cell cycle arrest, the alteration of the expression of cyclin E-Luc fusion protein was analyzed by immunoblotting (C), and the luciferase activity was measured at various time points (D). Meanwhile, the cell content (E) and cell cycle distribution (F) were analyzed by flow cytometer. All the groups have four replicates and the experiments were repeated for three times.

We fused luciferase protein to cyclin E, a tightly regulated cyclin that is expressed in the G1 phase and subsequently degraded during G1/S transition, to develop fluorescent probes that indicate whether individual live cells are in the G1 phase. First, an expression vector encoding the fusion protein of cyclin E linked to firefly luciferase under the control of cyclin E promoter was generated and named CycE-Luc (Figure 1A). As shown in Supplementary Figure S1A, the positive clone of CycE-Luc plasmid was digested by enzymes showing a striped band nearly 8000 bp in Pst I single enzyme lane, two bands near 7000 bp and 1200 bp in Hind III single enzyme lane, and three bands near 7000 bp, 700 bp and 500 bp in Hind III and Pst I double enzyme lane. DNA sequencing confirmed the formation of the CycE-Luc construct (Supplementary Figure S1C) and the chimeric protein was detected in HeLa cells under the control of the cyclin E promoter by immunoblot analysis (Figure 1B). These results demonstrated that the CycE-Luc fusion protein was efficiently expressed in mammalian cells.

### Expression of CycE-Luc fusion protein is regulated in a cell cycle-dependent manner

To investigate whether CycE-Luc is regulated in a cell cycle-dependent manner, HeLa cells stably expressing CycE-Luc were blocked with nocodazole and then released into the cell cycle. Subsequently, cells were harvested for the luciferase activity assay or flow cytometric analysis at various post-release time points. Determination of the DNA content by flow cytometry revealed that the expression of CycE-Luc fusion protein (Figure 1C) and luciferase activity (Figure 1D) were the lowest at 0 h, increased to the highest (18×10^4^) during 3 to 9 h release time when cells entered the G1 phase, and then reduced when cells entered into S and G2/M phases (Figure 1E and 1F).

### Establishment of CycE-Luc2 monoclonal HeLa cells for highly sensitive monitoring of the G1 phase

**Figure 2:**
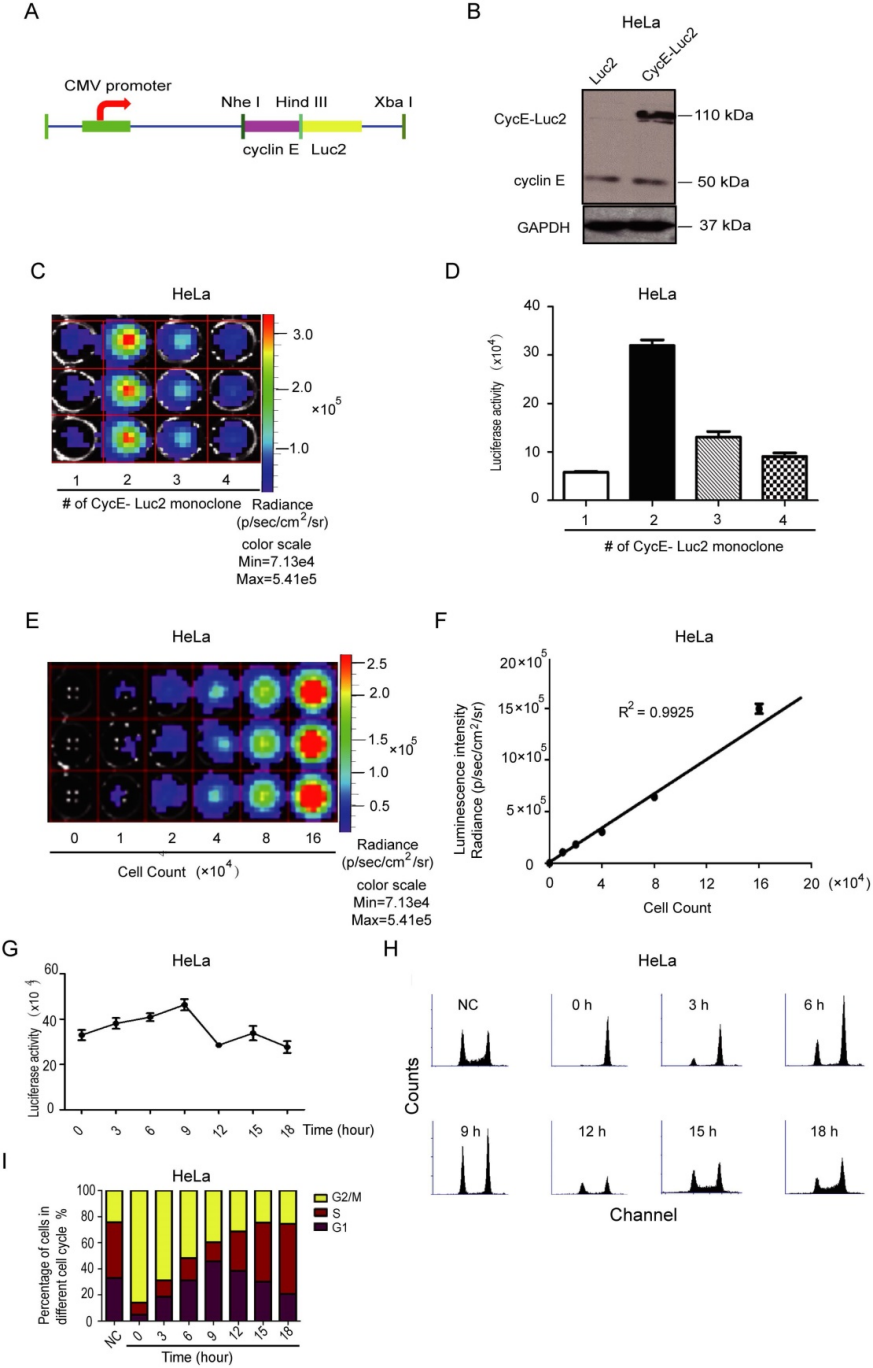
** Characterization of bioluminescent CycE-Luc2 reporter for the G1 phase in HeLa cells.** (A) Schematic diagrams of CycE-Luc2 constructs. The recombinant plasmid CycE-Luc2 encoding cyclin E cDNA fused in-frame at the N termini of luciferase 2 cDNA under the control of the CMV promoter. (B) CycE-Luc2 fusion protein was expressed in transfected HeLa cells by immunoblotting. (C&D) Different clones of stable expressed CycE-Luc2 was established and imaged using the IVIS Lumina imaging system to obtain FLUX measurements (C) and quantitated (D). (E&F) HeLa-CycE-Luc2 clone 2 cells were serially diluted, placed into wells of a 24 well plate, and immediately imaged using the IVIS Lumina imaging system to obtain FLUX measurements (E), and a plot comparing total flux to cell number was generated (F). (G-I) HeLa-CycE-Luc2 clone 2 cells were arrested in G2/M phase by growth in Nocodazale (0.4 µg/mL L). After removing Nocodazale to release G2/M cell cycle arrest, the alteration of the luciferase activity was measured at various time points (G). Meanwhile, the cell content (H) and cell cycle distribution (I) were analyzed by flow cytometer and quantitated. All the groups have four replicates and the experiments were repeated for three times. Error bars indicate standard error.

As shown in Figure 1, due to the low cyclin E promoter activity, the luciferase signal of CycE-Luc was relatively low. To increase the abundance of this reporter, we constructed another expression vector encoding a fusion protein of cyclin E linked to firefly luciferase 2 under the control of CMV promoter, named CycE-Luc2 (Figure 2A and Supplementary Figure S1B). As displayed in Figure 2B, Western blotting showed that CycE-Luc2 fusion protein was at a size of 110 kDa similar to CycE-luc. Through 96 orifice screening, 4 clones of stable CycE-Luc2-expressing HeLa cell lines were established of which the biological fluorescence signal intensity of clone 2 was significantly higher than the other 3 clones (Figure 2C and 2D). When CycE-Luc2 HeLa clone 2 was serially double diluted from 16×10^4^ cells (Figure 2E), the luciferase activity was correspondingly decreased with a high correlation between the intensity of the bioluminescent signal and the cell number (R^2^=0.9925, Figure 2F). Importantly, the luciferase activity in CycE-Luc2 HeLa clone 2 cells increased from 3 to 9 h release-time after nocodazole blocking (Figure 2G). Flow cytometry analysis showed that during this period, the cells entered the G1 phase (Figure 2H and 2I), suggesting the successful establishment of a sensitive CycE-Luc2 reporter for monitoring the G1 phase.

### CycE-Luc2 reporter monitors the G1 phase arrest in MCF-7 breast cancer cells *in vitro*

**Figure 3:**
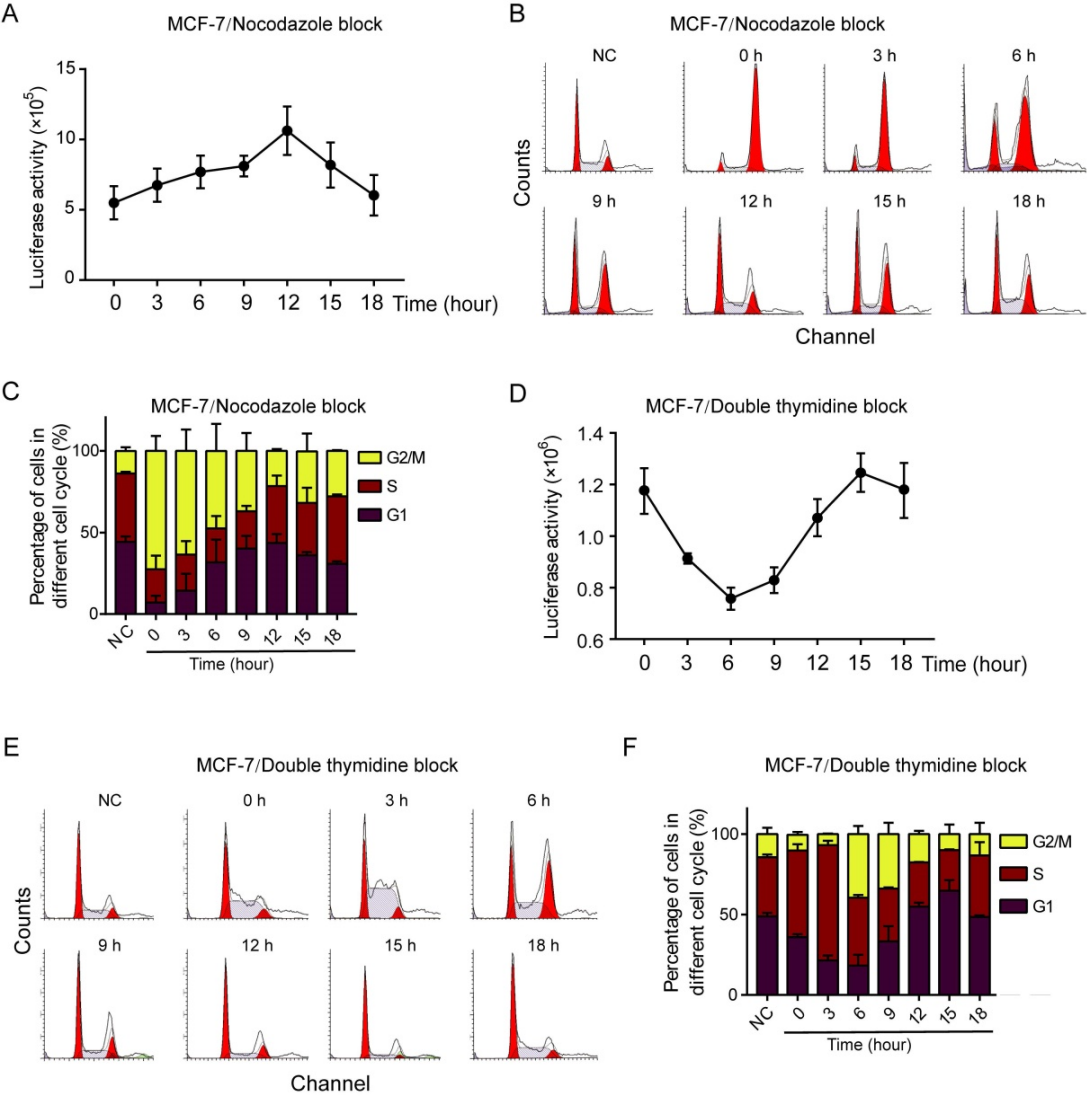
** Characterization of CycE-Luc2 reporter for the G1 phase in MCF-7 breast cancer cells.** CycE-Luc2 plasmid was transiently transfected in MCF-7 cells. Then, CycE-luc2 expressing MCF-7 cells were synchronized by either 0.4 µg/mL nocodazole (A-C) or 2 mmol/L thymidine treatment (D-F). Later, the luciferase activity was measured at different time points (A, D). The change of cell content (B, E) and cell cycle distribution (C, F) were analyzed by flow cytometer. All the groups have four replicates and the experiments were repeated for three times. Error bars indicate standard error.

To test if CycE-Luc2 reporter could monitor cell cycle change in other cells, an ER positive breast cancer cell line MCF-7, which is more sensitive to CDK4/6 inhibitors, was applied. CycE-Luc2 overexpressed MCF-7 cells were synchronized with either nocodazole or double thymidine blocking. When cells were blocked with nocodazole, the luciferase activity was the lowest (4.4×10^6^), and increased steadily with the highest at 12 h (8.49×10^6^) after release (Figure 3A). Flow cytometry analysis verified that the cells was blocked at G2/M phase and re-entered the G1 phase after release (Figure 3B and 3C). When blocked with double thymidine method, MCF-7-CycE-Luc2 cells showed higher luciferase activity (1.17×10^6^) at 0 h, and decreased to the lowest (0.76×10^6^) at 6 h after release, then increased eventually thereafter (Figure 3D). CycE-Luc2 fusion protein expression detected by Western was consistent with luciferase activity and cell cycle distribution (Supplementary Figure S2A, S2B). Flow cytometry analysis demonstrated that the cells entered the S and G2/M phases from 0 to 6 h after release, and cells entered G1/S phase from 6 to 12 h (Figure 3E and 3F). All these results confirmed that the CycE-Luc2 was a sensitive reporter for monitoring the G1 phase in breast cancer cells.

### CycE-Luc and CycE-Luc2 degradation is mediated by the ubiquitination-proteasome system

**Figure 4:**
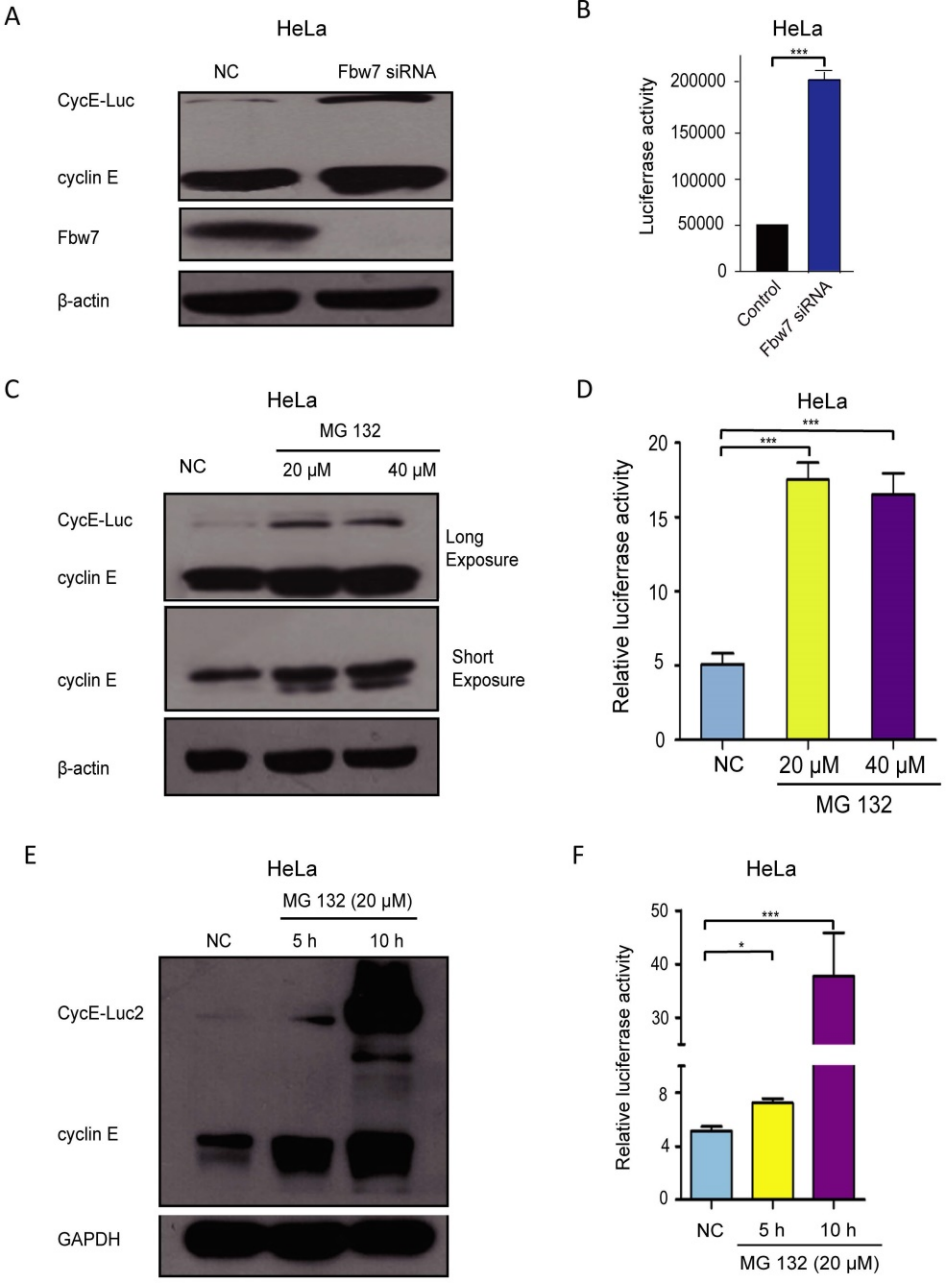
** The degradation of CycE-Luc or CycE-Luc2 was mediated by proteasome proteolysis.** (A and B) HeLa-CycE-Luc cells were transiently transfected with either Fbw7 siRNA or mock NC siRNA, cells lysed were used for analyzing CycE-Luc protein expression (A) and luciferase activity (B). (C and D) After HeLa-CycE-Luc cells were treated with the proteasome inhibitors MG132 (20 µm and 40 µm) for 6 hours. Cells lysates were used for analyzing the expression of CycE-Luc fusion protein by immunoblotting (C) and luciferase activity (D). (E and F) After HeLa-CycE-Luc2 clone 2 cells were treated with the proteasome inhibitors MG132 (20 µm) for 5 or 10 hours. Cells lysates were used for analyzing the expression of CycE-Luc2 fusion protein by immunoblotting (C) and luciferase activity (D). All the groups have four replicates and the experiments were repeated for three times. Error bars indicate standard error. *: p<0.05; ***: p<0.001.

Previous research had shown that SCF/Fbw7 mediates degradation of cyclin E 28. To investigate whether SCF/Fbw7 regulated the turnover of CycE-Luc, it was depleted from CycE-Luc cells using SCF/Fbw7 siRNA. Forty-eight hours after siRNA transfection, Fbw7 was effectively depleted (Figure 4A). The expression of CycE-Luc was significantly increased as compared with mock-transfected cells by the immunoblot assay as well as luciferase activity analysis (Figure 4A and 4B). We further identified whether CycE-Luc was eliminated via proteasome degradation. After HeLa cells were treated with the proteasome inhibitor MG132, we observed a significant increase in CycE-Luc protein as well as the luciferase activity (Figure 4C and 4D). The degradation of CycE-Luc2 was also controlled by the proteasome complex (Figure 4E and 4F). Thus, these results revealed the degradation of CycE-Luc and CycE-Luc2 was mediated via the proteasome complex.

### CycE-Luc reporter can monitor cell cycle inhibition by 5-FU *in vitro* and *in vivo*

**Figure 5:**
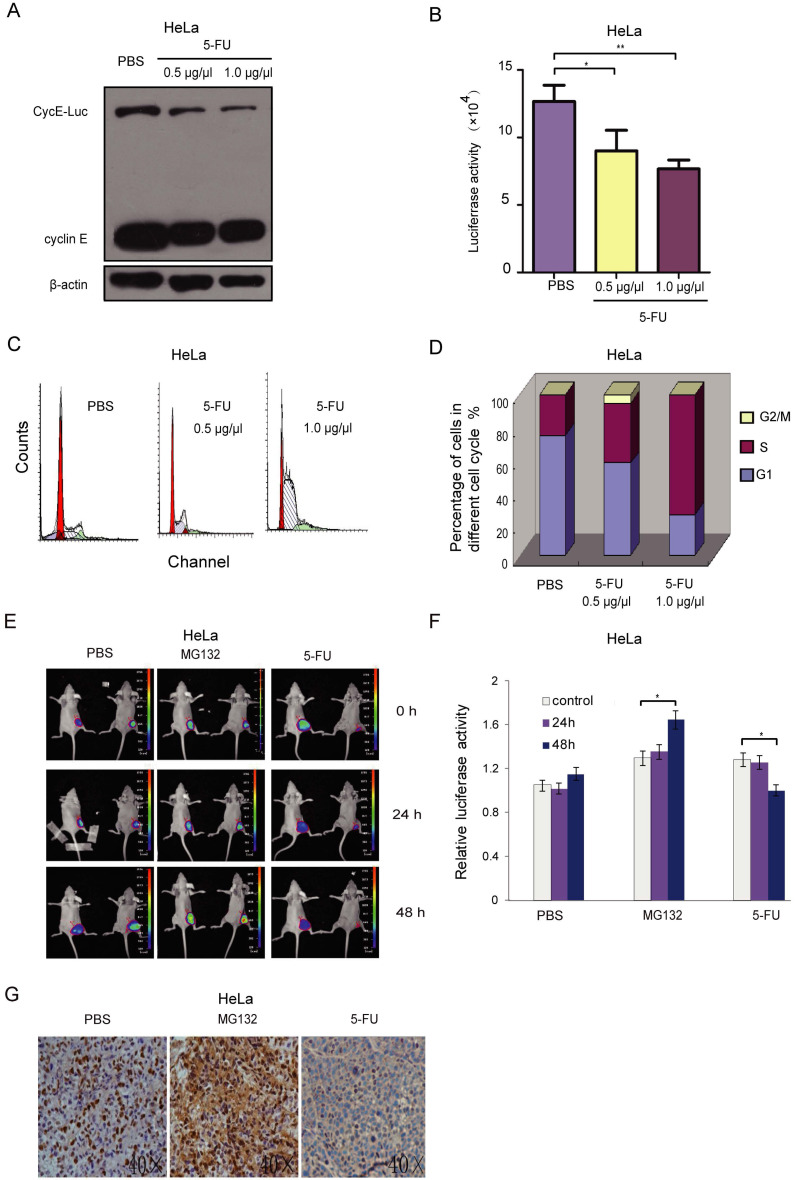
** Bioluminescence imaging of CycE-Luc reporter for monitoring antitumor efficacy of 5-FU *in vitro* and *in vivo*.** (A-D) After treatment with 5-FU (0.5 µg/µl and 1 µg/µl) for 20 h, the expression of the reporter and bioluminescence imaging of HeLa-CycE-Luc cells were analyzed by immunoblotting (A) or assayed for luciferase activity (B), respectively. Meanwhile, the change of cell content (C) and cell cycle distribution (D) by 5-FU treatment were analyzed by flow cytometer. All the experiments repeated three times. Error bars indicate standard error. (E-G) HeLa cyclin E-Luc cells were injected subcutaneously into the right flank of BALB/C nude mice. When tumor nodule was about 100 mm^3^, PBS (n=5), MG132 (2 mg/kg per mouse, n=5) or 5-FU (25 mg/kg per mouse, n=5) was i.p. injected into nude mice, the bioluminescent images (E) were obtained 0, 24 and 48 h after injection, and relative induction folds of bioluminescent signal was calculated and normalized (F). Finally, 48 h after 5-FU treatment, tumor mass was removed and paraffin embedded sections were stained with cyclin E antibody (G). All the groups have four replicates and the experiments were repeated for three times. Error bars indicate standard error. *: p<0.05; **: p<0.01.

To evaluate the effect of the anti-tumor drug on cell cycle using this reporter, stably CycE-Luc expressing cells were treated with 5-FU at a dose of 0.5 g/L and 1.0 g/L. Cells were collected for analysis of relative luciferase activity and immunoblotting after 20 h incubation with 5-FU. The CycE-Luc level as well as the luciferase activity was significantly decreased after 5-FU treatment in a dose-dependent manner (Figure 5A and 5B). The 5-FU treatment also resulted in increased accumulation in S-phase and decreased accumulation in the G1 phase in a dose-dependent manner (Figure 5C and 5D). Thus, CycE-Luc reporter system could be used to monitor the antitumor efficacy of 5-FU. Next, we evaluated the effect of 5-FU on the cell cycle using bioluminescent CycE-Luc reporter in a subcutaneous mouse tumor model. First, HeLa-CycE-Luc cells were implanted subcutaneously in nude mice. Three weeks after implantation, the tumor nodules became noticeable and were imaged using the Xenogen IVIS Lumina imaging system. After 48 h treatment of tumors with 5-FU (25 mg/kg per mouse, n=5), the bioluminescent signal of CycE-Luc was significantly decreased as compared to the increased signal observed by MG132 treatment (2 mg/kg per mouse, n=5) and no change in the PBS control group (Figure 5E and 5F). To assess whether the increase in bioluminescence activity was concomitant with the expression of CycE-Luc, the level of cyclin E was determined by immunohistochemistry. The results showed that MG132 treatment significantly increased and 5-FU treatment significantly decreased cyclin E protein levels in the tumor tissue (Figure 5G). Thus, these results demonstrated that the CycE-Luc reporter was suitable for monitoring the cell cycle status affected by anti-tumor drugs in a dynamic, non-invasive way.

### CycE-Luc2 reporter can monitor G1-phase arrest by palbociclib treatment *in vitro* and* in vivo*

**Figure 6:**
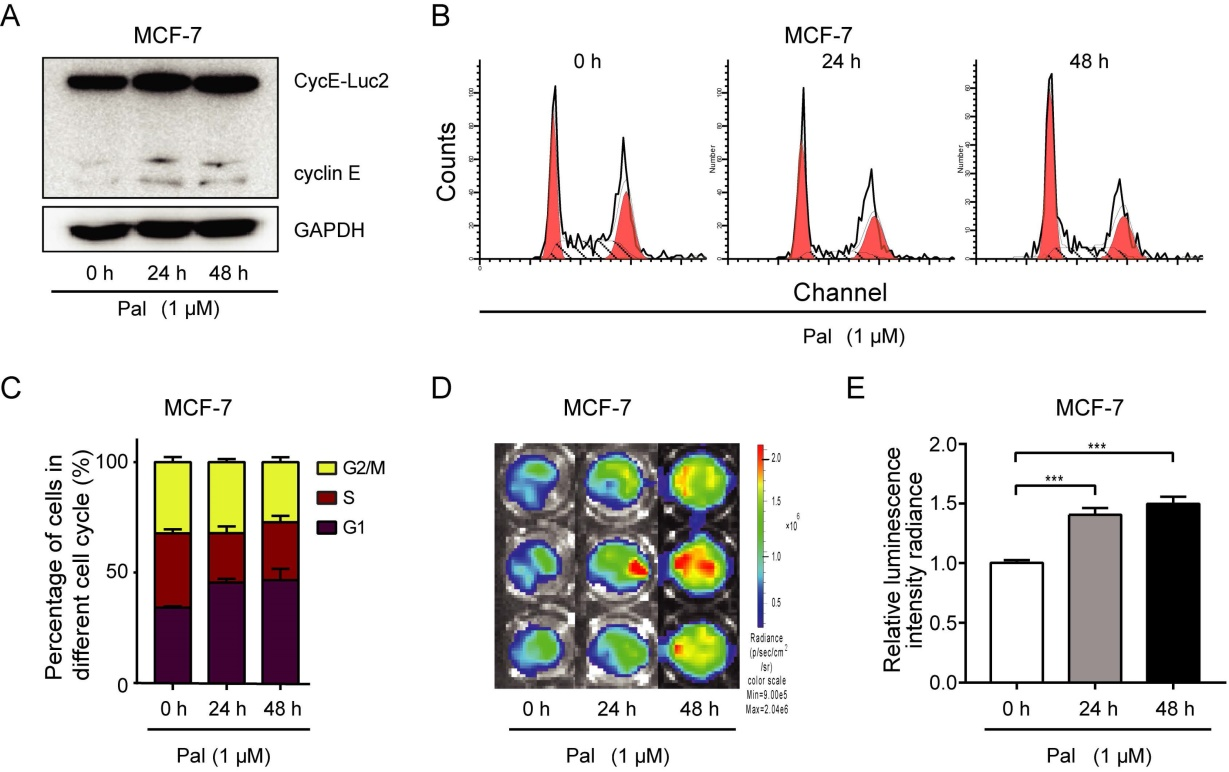
** Monitoring G1-phase arrest by palbociclib using CycE-Luc2 reporter in MCF-7 breast cancer cells.** MCF-7 cells were transiently transfected with CycE-Luc2 reporter. Later, cells were treated with palbociclib (Pal, 1 µM) for 24 or 48 h. Cells lysates were used for analyzing the expression of CycE-Luc2 fusion protein by immunoblotting (A). The cell content (B) and cycle distribution (C) were analyzed by flow cytometer. The luciferase activity of CycE-Luc2 was measured by bioluminescence imaging (D). (E) Quantitative analyses of average luminescence intensities of D. All the groups have four replicates and the experiments were repeated for three times. Error bars indicate standard error. ***: p<0.001.

Palbociclib, a highly selective CDK4/6 inhibitor, which blocks the replication cycle and proliferation of tumor cells, is a promising anticancer drug for breast cancer patients. MCF-7 cells transiently transfected with CycE-Luc2 were treated with palbociclib for 24 h, and palbociclib at 100 nM or above caused G1 arrest (Supplementary Figure S3). Palbociclib treatment also increased cycE-Luc2 detected by luciferase assay and *in vitro* imaging. MCF-7-CycE-Luc2 cells treated with 1 μM palbociclib for 24 h or 48 h, and we demonstrated an increase of CycE-Luc2 in a time dependent manner (Figure 6A). There results were consistent with G1 phase arrest (Figure 6B, 6C). Moreover, the luciferase activity was also increased in a time-dependent manner (Figure 6D, 6E).

**Figure 7:**
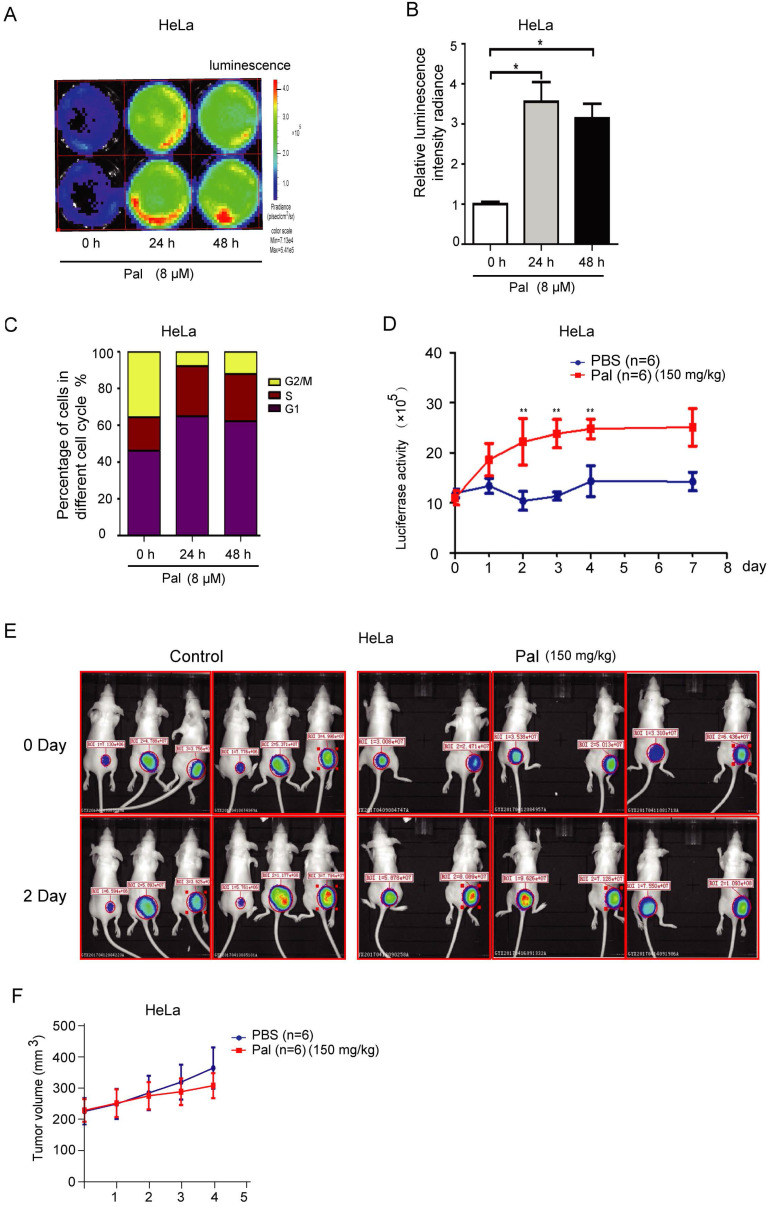
** Bioluminescence imaging of HeLa-CycE-Luc2 cells after palbociclib treatment* in vitro* and* in vivo.*** (A-C) After treatment with palbociclib (Pal, 8 µM) for 24 and 48 h, the HeLa-CycE-Luc2 cells were imaged using the IVIS Lumina imaging system to obtain FLUX measurements (A) and analyzed for the effect on luciferase activity (B). Meanwhile, the cell cycle distribution (C) by Pal treatment were analyzed by flow cytometer. All the experiments repeated three times. Error bars indicate standard error. *: p<0.05. (D & E) HeLa cyclin E-Luc cells were injected subcutaneously into the right flank of BALB/C nude mice. When tumor nodule was about 100 mm^3^, PBS and Pal (150 mg/kg per mouse, n=6 each group) was i.p. injected into nude mice, the bioluminescent images were obtained once a day for 8 days and analyzed (D), the original bioluminescent images of 0 and 2 days Pal treatment were represented (E), and the tumor volume growth measured for 4 days (F). *: p<0.05; **: p<0.01.

To further explore whether CycE-Luc2 reporter could monitor G1-phase arrest by palbociclib *in vitro* and *in vivo*, HeLa-CycE-Luc2 clone 2 cells were used to perform *in vitro* and* in vivo* experiment. Luciferase activity analyses on synchronized cells revealed a significant 3- to 4- fold increase in bioluminescent signal of HeLa-CycE-Luc2 clone 2 cells after 24 or 48 h incubation with 8 μM palbociclib (Figure 7A and 7B). Furthermore, flow cytometric analyses showed that cells arrested in the G1-phase after 24 or 48 h incubation with palbociclib (Figure 7C). To validate the ability of CycE-Luc2 reporter for monitoring G1-phase arrest by palbociclib in an* in vivo* model. Implanted HeLa-CycE-Luc2 clone 2 cells were subcutaneously injected into nude mice. Three weeks after implantation, the mice were treated with palbociclib (150 mg/kg) and imaged using the Xenogen IVIS Lumina imaging system once a day. Our results showed that the bioluminescence signals from HeLa-CycE-Luc2 tumors increased significantly in response to palbociclib (Figure 7D and 7E), while the tumor volume has no significant difference between palbociclib and PBS group (Figure 7F).

## Discussion

Bioluminescence reporter proteins have been widely used in the development of tools for monitoring biological events in living organisms in real-time 19. Many aspects of drug development can be facilitated using bioluminescence reporters as an indicator to discover new targets, identify novel drug candidates, and validate their potency 29. In the present study, we developed two optical reporters consisting of genetically encoded cyclin E fused to luciferase (Luc or Luc2), which reflected the change in G1 phase of the cell cycle. We then used the two G1 phase reporters to evaluate cell cycle-specific anti-cancer drugs, 5-FU and palbociclib,* in vitro* and *in vivo* using the IVIS imaging system and demonstrated their ability to provide the pharmacodynamic readout of cell cycle-specific anti-cancer effect. We showed that these fusion proteins accumulated in the G1-phase and were degraded during S/G2/M phase similar to endogenous cyclin E. Importantly, altered luciferase activity of the reporters matched with cell cycle progression, indicating their potential application for monitoring the response to cell cycle-specific anti-cancer drugs.

As the bioluminescence signal is amplifiable through an enzymatic reaction, these optical reporters can be applied to cell lysates, live cells in culture, and in small animals at limited depths. For example, p27-luciferase-expressing tumor cells were used to monitor CDK2 activity *in vivo* 25, and stably NFκB-responsive element-luciferase-expressing tumor cells were evaluated to monitor the response of LPS or TNF-α stimulation in mice 18. In the present study, we generated two constructs driven by either native cyclin E or artificial CMV promoter. In cyclin E promoter driven reporter, luciferase expression is too weak to perform *in vivo* study. We observed high luciferase activity in the CMV promoter driven CycE-Luc2 reporter, and further demonstrated cell cycle dependent regulation of CycE-Luc2 protein, especially, its degradation through ubiquitination dependent manner. Especially, when we employed these reporters in an *in vivo* setting, we observed that the CycE-Luc2 reporter provided a stronger bioluminescent signal than CycE-Luc. Thus, the high sensitivity, simplicity, cost-effectiveness, and wide dynamic range of bioluminescent reporters are valuable features for drug-screening, toxicological testing, and therapeutic response, making them highly attractive for the development of novel anticancer drugs.

Directly targeting the aberrant cell-cycle progression represents a potential therapeutic modality such as chemotherapy and radiotherapy. In this respect, over the past two decades, efforts have been focused on developing inhibitors of the cyclin-dependent kinases (CDKs). Active kinase complexes of CDK4/6-D-type cyclins promote G1/S phase transition through phosphorylation of Rb, p107, and p130 30, 31. Palbociclib was reported in 2004 as a specific, high affinity inhibitor of CDK4 and 6 with no appreciable activity against 36 additional kinases 32. Treatment with palbociclib led to profound G1-phase arrest and cytostasis, especially in mice bearing human breast and colorectal xenografts. Oral administration of palbociclib has been shown to induce tumor regression with inhibition of RB phosphorylation 32, 33. In subsequent randomized trials, palbociclib, in combination with endocrine therapy, has been reported to prolong progression-free survival 5, 7, 8, leading to its approval in patients with metastatic HR-positive/HER2-negative breast cancer 34. In the clinics, except RECIST as a standard method to assess the effect of anti-cancer drugs, 3′-deoxy-3′-[^18^F]fluorothymidine (^18^F-FLT) and [^18^F]fluoro-2-deoxy-D-glucose (^18^F-FDG) positron-emission tomography (PET) imaging provides a non-invasive approach to monitor the early response to palbociclib. Decreased ^18^F-FLT accumulation and S-phase depletion after palbociclib treatment were observed in MCF-7 cells and MCF-7 xenografts 35. Also, several clinical trials of surrogate markers for palbociclib verified that early decrease of RB phosphorylation and Ki67 correlated with the effect of the drug on cell proliferation 36, 37. Recently, ^18^F- palbociclib have been developed as a promising positron emission tomography (PET) imaging agent for CDK4/6 activation in MCF-7 xenograft 38. Thus, the molecular imaging methods for monitoring the alteration of molecular targets of palbociclib allow fast evaluation of drug efficacy and minimize its toxicity, ultimately reducing cost.

In the present study, based on our understanding of molecular events that follow palbociclib treatment, we have developed a molecular imaging method to monitor the drug response using CycE-Luc2 reporter. Intriguingly, G1-phase arrest caused by palbociclib, as evidenced by the induction of bioluminescence, correlated with flow cytometry result. In particular, our experiments *in vivo* showed that bioluminescence can circumvent tissue auto-luminescence resulting in a better signal to noise ratio. The luciferase signal increased at 24 h, and reached peak levels lasting up to 96 h. It is clear that the development of these reporters opens up the possibility of monitoring the pharmacological effects of G1 phase-specific anti-cancer drugs. Thus, this G1 phase-specific reporter has provided evidence for its potential use to determine the fraction of tumor cells in the G1 phase after treatment with palbociclib. Bioluminescence imaging has its ability to enhance the analysis of drug response in the patient-derived xenografts (PDX) 39. Thus, PDX could also be engrafted into our model to open new avenues for developing personalized therapeutic approaches in the future. However, these imaging biomarkers need to be extensively evaluated, particularly for late-stage clinical studies, to determine whether they can be used as primary measures of the effectiveness of a drug and translated into a clinically meaningful benefit. Currently, there are hardly any FDA-accepted imaging biomarkers, and especially there are no validated imaging-based surrogate endpoints.

## Concluding remarks

CDK4/6 is required for cell cycle entry, and is also attractive target for new developments in cancer therapeutics. The clinical use of CDK4/6 inhibitors require a robust validation for physiological and metabolic treatment response of those drugs by obtaining quantitative imaging endpoints from PET, MRS, and/or optical imaging platforms 40. Novel G1 phase reporters (cycE-Luc and cycE-Luc2) were successfully applied for molecular and functional imaging of cell cycle-specific agents to elucidate their biological activity and drug-target effects both *in vitro* and *in vivo.* They are also useful to investigate basic processes of cell cycle regulation and their modulation by intracellular pathways and receptor signaling. Our results suggest that cyclin E-Luc reporters might serve as a clinically translatable approach for predicting and monitoring response to CDK4/6 inhibitors in patients with ER-positive breast cancer. Nevertheless, the evaluation of these optical reporters with optimized properties is still of outstanding interest for monitoring early response of cell cycle-specific drugs in patients who are more likely to benefit from these drugs.

## Supplementary Material

Supplementary figures and tables.
