# Supplementary material for: The G1 phase optical reporter serves as a sensor of CDK4/6 inhibition in vivo
Source: Int J Biol Sci. 2021 Jan 31;17(3):728–41. doi: 10.7150/ijbs.52101 (PMC7975702; doi:10.7150/ijbs.52101)
Supplement: Supplementary file 1 — Supplementary figures and tables. [file ijbsv17p0728s1.pdf]

Supplementary Fig.S1. Generation of Cyclin and luciferase fusion protein expressed vectors. The plasmids for CycE-Luc (A) and CycE-Luc2 (B) was digested by enzymes and run on an agarose gel. (C) The sequence of CycE-Luc plasmid was verified by an automated DNA sequencer.

Supplementary Fig.S2. The expression of CycE-Luc2 fusion protein was analyzed by immunoblotting. CycE-luc2 plasmid was transiently transfected in MCF-7 cells. Then, CycE-luc2 expressing MCF-7 cells were synchronized by either nocodazole (A) or thymidine treatment (B), and the expression of CycE-Luc2 fusion protein was detected by immunoblotting, respectively.

Supplementary Fig.S3. Monitoring G1 arrest by different doses of palbociclib using CycE-Luc2 reporter in MCF-7 breast cancer cells. MCF-7 cells transiently transfected with CycE-Luc2 fusion protein were treated with different doses of palbociclib (0, 10, 100 or 1000 nM) for 24h. Cells lysates were used for analyzing the expression of CycE-Luc2 fusion protein by immunoblotting (A). The cell content (B) and cycle distribution (C) were analyzed by flow cytometer. The luciferase activity of CycE-Luc2 was measured by bioluminescence imaging (D). (E) Quantitative analyses of average luminescence intensities of D. All the groups have four replicates and the experiments were repeated for three times. Data are expressed as mean ± SD.*: $p<0.05$； **: $p<0.01$.

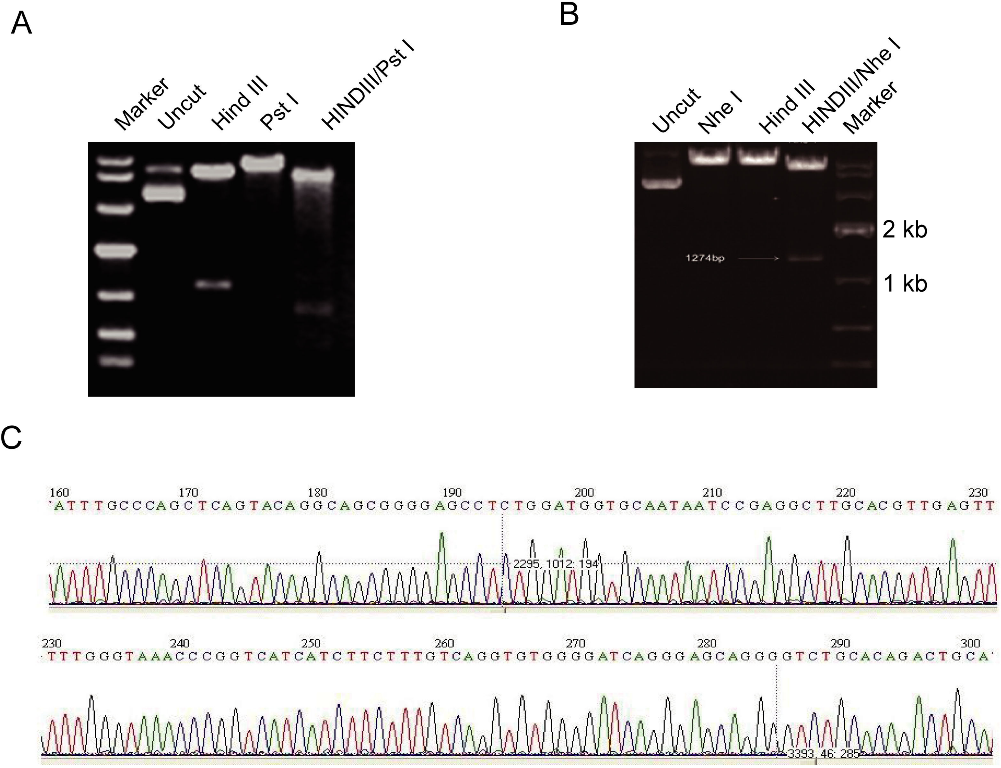

Supplmentary Figure S1

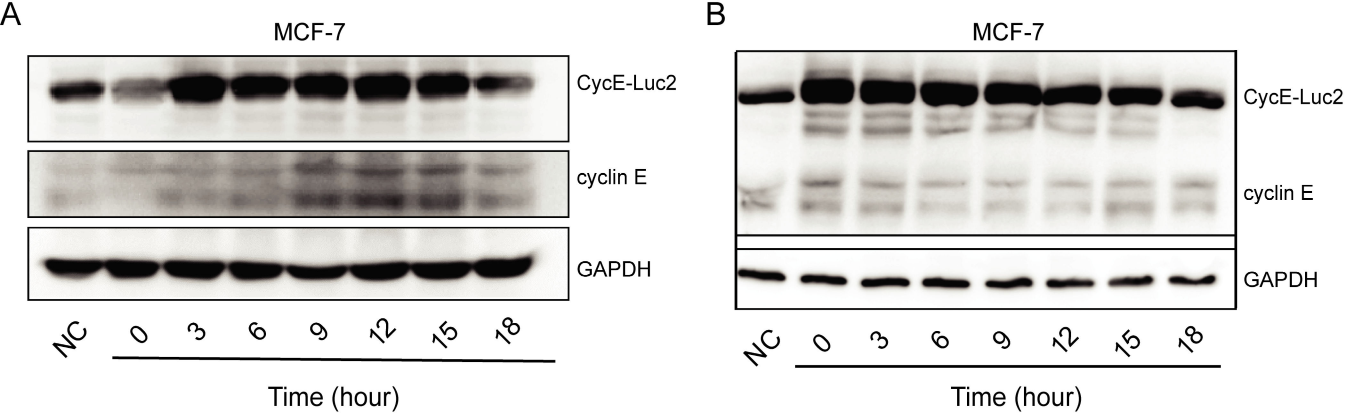

Supplmentary Figure S2

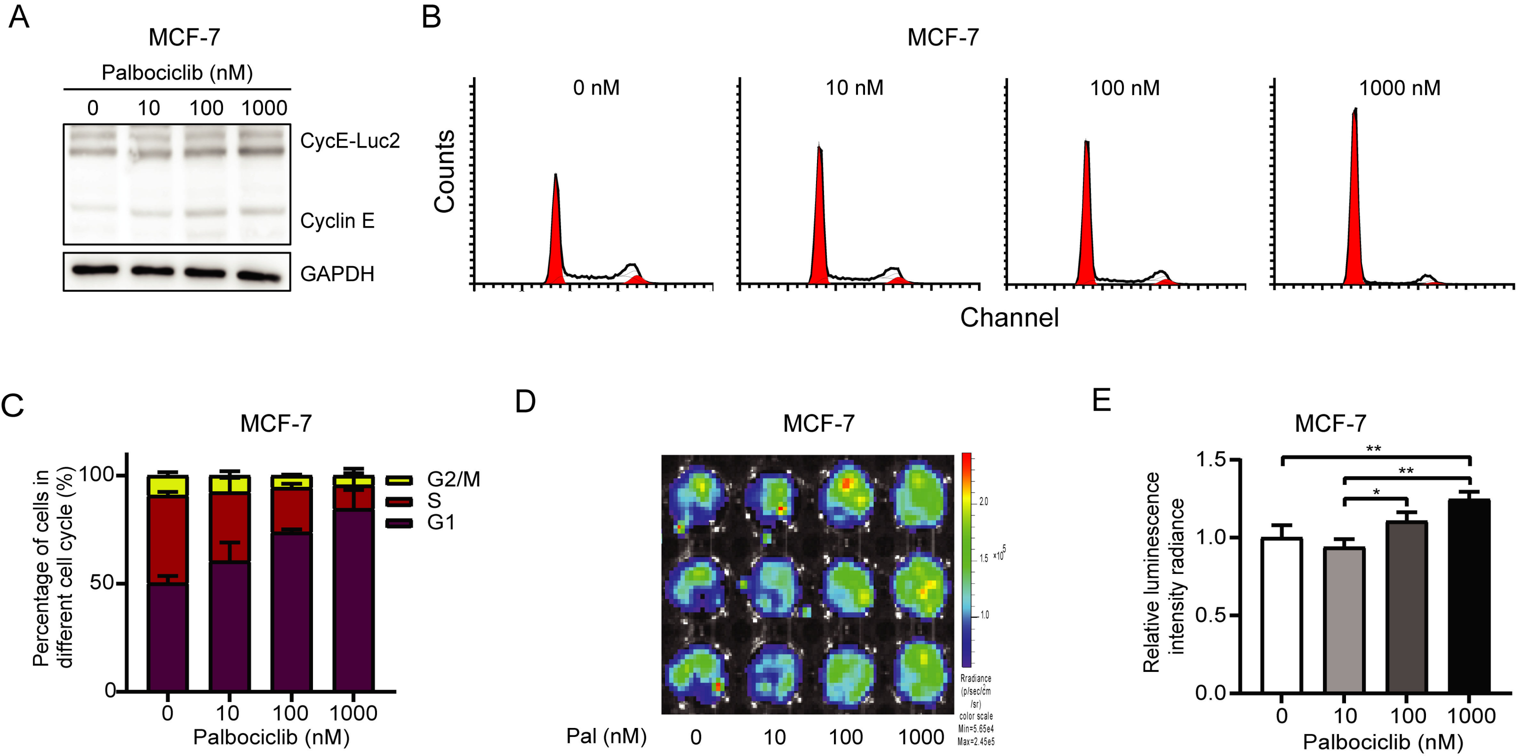

Supplmentary Figure S3
